# Synchronous occurrence of gastrointestinal stromal tumors and other digestive tract malignancies in the elderly

**DOI:** 10.18632/oncotarget.3108

**Published:** 2015-03-07

**Authors:** Chaoyong Shen, Haining Chen, Yuan Yin, Jiaju Chen, Luyin Han, Bo Zhang, Zhixin Chen, Jiaping Chen

**Affiliations:** ^1^ Department of Gastrointestinal Surgery, West China Hospital, Sichuan University, Chengdu 610041, Sichuan, China; ^2^ Intensive Care Unit, West China Hospital, Sichuan University, Chengdu 610041, China

**Keywords:** Gastrointestinal stromal tumors, Synchronous neoplasm, Digestive tract malignancies, Elderly

## Abstract

**Background/Aims:**

Elderly patients with gastrointestinal stromal tumors (GISTs) synchronous with other digestive tract malignancies have been rarely reported. In this study, clinicopathological characteristics were evaluated among elderly patients with GISTs with or without coexisting digestive tract malignancies.

**Methods:**

A total of 161 patients (≥65 years) were retrospectively reviewed at the West China Hospital, Sichuan University from January 2009 to June 2014.

**Results:**

Sixty-one patients were diagnosed with synchronous digestive tract malignancies (synchronous group), whereas 100 patients were diagnosed with no synchronous condition (no-synchronous group). The synchronous group exhibited a higher percentage of males (70.49% vs. 53.00%, *P* = 0.028) and poorer Eastern Cooperative Oncology Group performance status than the no-synchronous group (*P* = 0.029). The three-year overall survival (OS) rate was significantly lower among patients with synchronous digestive tract malignancies than that among patients without synchronous condition (64.5% vs. 84.0%, *P* = 0.003). Multivariate analysis showed that the presence of synchronous digestive tract malignancies (*P* = 0.002), co-morbidity (*P* = 0.004), and mitotic count ≥10 mitoses/50 high power fields (*P* = 0.012) were associated with poor OS.

**Conclusions:**

A synchronous condition with other digestive tract malignancies is common in elderly patients with GISTs. OS primarily depends on synchronous digestive tract malignancies, mitotic count, and co-morbidity.

## INTRODUCTION

Gastrointestinal stromal tumors (GISTs) are rare mesenchymal tumors found within the abdominal cavity (mainly located in the stomach and small intestine and occasionally in the mesentery, retroperitoneum, esophagus, and omentum) with an estimated incidence of 10 to 20 per million [1–3]. The median age at the time of GIST diagnosis is 63 years [4]. Gastric GISTs are extremely rare among the elderly, as described in the literature [5]. Microscopic GISTs (smaller than 1 cm) can be incidentally found during endoscopy or surgery of other malignancies or during post-operative pathological examination of resected surgical specimens other than GISTs; these GISTs are detected in approximately 20% to 35% of the elderly [6, 7].

The coexistence of GISTs with other malignant tumors is complex. This subject has gained considerable attention relative to clinical management and surgery, particularly in the area of personalized medicine. Most GISTs detected through surgery of other malignancies are small and asymptomatic. However, few of these tumors may be nonmalignant when diagnosed but exhibit potential for malignant transformation. Furthermore, small GISTs with low mitotic activity occasionally demonstrate distant metastasis [7, 8]. This finding is contrary to the conclusion of Rossi [6], who reported that microGISTs are self-limiting lesions. Thus, therapies for GISTs synchronous with other malignancies have not been well documented and have been debated for many years.

Numerous patients with GISTs synchronous with other neoplasms have been described, but most reports present single case studies [9–11] or small samples [3, 12–14]. Hence, the clinical management, histopathological features, and prognostic predictors remain unknown. To our knowledge, no study has specifically targeted elderly patients with GISTs synchronous with other digestive tract malignancies. Therefore, we aimed to determine the clinicopathological characteristics and treatment and to evaluate prognostic factors on the basis of the data obtained from 161 elderly patients with GISTs who were consecutively admitted in our institution.

## RESULTS

### Patient demographics and clinical characteristics

**Figure 1:**
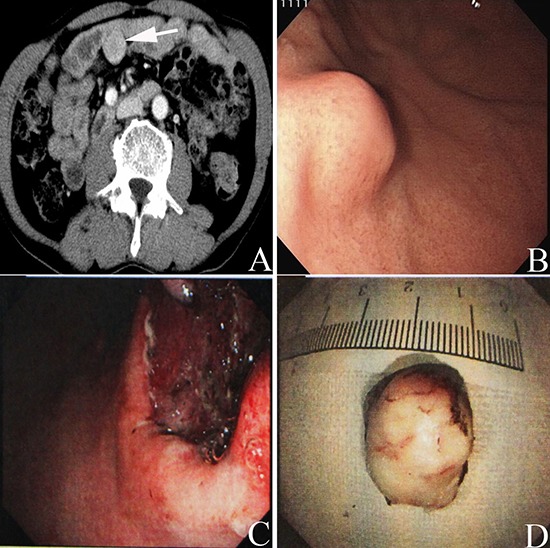
Preoperative abdominal enhanced CT scan showed a mass (white arrow in A) with a size of 3.5cm×3 cm located in the abdominal cavity in one patient with rectal cancer Upper gastrointestinal endoscopy presented an intramural lesion **(B)** located in the body of the patient with GIST of the stomach synchronous with a small carcinelcosis located in the antrum of the stomach. Tumor complete resection was obtained through the endoscopic view **(C)** Image of the tumor resected through endoscopic resection; the tumor size was lower than 2 cm **(D)**.

The entire cohort comprised 161 elderly patients with GISTs and included 96 (59.6%) males and 65 (40.4%) females with a mean age of 69.93±4.37 years and a median age of 69 years (range, 65–84 years). Of these 161 enrolled patients, 61 were diagnosed with GISTs synchronous with other digestive tract malignancies (synchronous group), whereas 100 were diagnosed with no-synchronous condition (no-synchronous group). The most common localization site of GISTs was the stomach (*n* = 134, 83.2%), followed by the small intestine (*n* = 17, 10.6%) and other parts of the system (*n* = 10, 6.2%; including mesentery of small intestine, omentum, rectum, and transverse mesentery). A high number of GISTs located in the stomach were observed in the synchronous group (*P* = 0.005). The synchronous group also presented a higher percentage of males (70.49% vs. 53.00%, *P* = 0.028) and poorer ECOG performance status (*P* = 0.029) than the no-synchronous group. Moreover, no statistical significance was detected with regard to age, co-morbidity, and hospital stay between the groups (*P* > 0.05). In the no-synchronous group, patients with GISTs variably presented abdominal discomfort/pain (*n* = 43), gastrointestinal bleeding (*n* = 36), and mass (*n* = 11), which were incidentally discovered for other reasons (*n* = 10). In the synchronous group, all but three patients (one case was preoperatively discovered through CT and two cases through electronic endoscopy; Figure 1AB) with GISTs were incidentally detected during surgery or postoperative pathologic examination. Data are shown in Table 1.

**Table 1:** Demographic and clinicopathologic data on 161 elderly patients with GISTs

Variables	Synchronous Group (*n* = 61, %)	No-synchronous Group (*n* = 100, %)	*P* value
Age, y	70.41±4.61	69.64±4.21	0.279
Gender			0.028
Male	43 (70.49)	53 (53.00)	
Female	18 (29.51)	47 (47.00)	
ECOG score			0.029
≤ 1	29 (47.54)	65 (65.00)	
≥ 2	32 (52.46)	35 (35.00)	
Tumor site			0.005
Stomach	58 (95.08)	76 (76.00)	
Small intestine	2 (3.28)	15 (15.00)	
Others^#^	1 (1.64)	9 (9.00)	
Tumor size, cm	1.02±0.86	6.59±4.48	<0.001
Mitotic count			<0.001
10 mitosis/50HPF	0 (0.00)	17 (17.00)	
<10 mitosis/50HPF	61 (100.00)	83 (83.00)	
NIH risk classification			<0.001
Very low and low	57 (93.44)	29 (29.00)	
Intermediate and high	4 (6.56)	71 (71.00)	
IM treatment			<0.001
Yes	1 (1.64)	27 (27.00)	
No	60 (98.36)	74 (74.00)	
IM duration, mo	8	8.22±1.06	-
Synchronous cancer^†^			
Esophageal carcinoma	28 (45.90)	-	
Gastric carcinoma	29 (47.54)	-	
Gastric lymphoma	1 (1.64)	-	
Rectal carcinoma	3 (4.92)	-	
Co-morbidity^&^			0.729
Present	23 (37.70)	35 (35.00)	
Absent	38 (62.30)	65 (65.00)	
Hospital stay, days	19.08±8.68	17.20±8.20	0.169

ECOG, Eastern Cooperative Oncology Group

# Others includes mesentery of small intestine, omentum, rectum and transverse mesentery

† Synchronous cancer refers to synchronous with digestive malignancies

& Co-morbidity includes chronic pulmonary disease, diabetes mellitus, cardiovascular and cerebrovascular disease, chronic liver and renal disease

HPF, High power field

NIH, National Institutes of Health

IM, Imatinib mesylate

### Tumor characteristics

Five patients presented liver or peritoneal metastasis at the time of diagnosis in the no-synchronous group. Sixty-one cases demonstrated GISTs synchronous with other digestive tract malignancies, with gastric carcinoma as the predominant (*n* = 29, 47.54%), followed by esophageal carcinoma (*n* = 28, 45.90%). In terms of TNM staging system, seven and six patients were diagnosed with stage I esophageal carcinoma and gastric carcinoma, respectively. Eight patients were classified with stage II esophageal carcinoma, and nine patients with stage II gastric carcinoma. Moreover, 13 and 12 patients were categorized into stage III esophageal carcinoma and gastric carcinoma, respectively. Finally, two patients were diagnosed with stage IV gastric carcinoma. Tumor diameter was statistically significant (*P* < 0.001). Patients in the no-synchronous group exhibited a larger tumor size than those in the synchronous group (6.59±4.48 cm vs. 1.02±0.86 cm, *P* < 0.001). Six patients (9.84%) presented a tumor size ≥3 cm, and 53 (86.89%) patients demonstrated no mitotic activity in the synchronous group. The majority of patients in the synchronous group presented lower mitotic count and higher percentage of very low and low risk than patients in of the no-synchronous group (*P* < 0.001). Patients with very low, low, intermediate, and high risk were detected in 4, 25, 22, and 49 and in 54, 3, 3, and 1 case in the no-synchronous and synchronous groups, respectively. Overall, 68 GISTs were found in the synchronous group (*n* = 61) because of multiple GISTs were detected in three patients, as summarized in Table 1.

### Surgery and IM treatment

A total of 158 patients (98.14%) underwent surgical resection for GISTs or resection of digestive tract malignancies coexisting with GISTs. All but five patients (two patients with palliative resection for gastric carcinoma and three patients with palliative resection for GISTs) underwent radical resection (R0 resection). Among these patients with surgical treatment, 10 underwent endoscopic resection performed by skilled endoscopic specialists, and R0 resection was obtained (Figure 1CD). The remaining three patients did not receive surgery. Two underwent IM preoperative therapy (partial responses were noted in these two cases), and one refused to receive any treatment (no-synchronous group) for unknown reasons. A total of 28 cases received IM therapy for the entire cohort with a median time of seven months (range, 1–19 months). However, IM dose reduction (200–300 mg/d) was required for 20 patients because of drug toxicity. Drug-related adverse effects were mainly classified as grades 1 and 2 and are presented in Table 2.

**Table 2:** Adverse events in the elderly GISTs patients. (*n* = 28)

Parameters	Grade 1/2 (%)	Grade 3/4 (%)
Edema	16 (57.14)	1 (3.57)
Anorexia	3 (10.71)	-
Hepatic function damage	5 (17.86)	1 (3.57)
Granulocytopenia	2 (7.14)	-
Nausea/vomiting	6 (21.43)	1 (3.57)
Anemia	4 (14.29)	-
Erythra	1 (3.57)	-
Muscle spasm	1 (3.57)	-
Asthenia	14 (50.00)	2 (7.14)
Ascites	-	1 (3.57)
Myalgia	3 (10.71)	-

### Tumor immunohistochemical features

A total of 164 (out of 168) GISTs were categorized as spindle type, 20 were epithelioid type, and two were mixed type. The following markers were positively expressed: CD117 (*n* = 146/168, 86.90%), CD34 (*n* = 138, 82.14%), S-100 (*n* = 13, 7.74%), smooth muscle actin (SMA; *n* = 39, 23.21%), desmin (*n* = 15, 8.93%), and Dog-1 (*n* = 154, 91.67%). No statistical significance was observed in the expression of CD117, CD34, S-100, SMA, desmin, and Dog-1 between the groups (*P* > 0.05). A total of 21 gene mutations were detected (16 mutations in KIT exon 11, three mutations in KIT exon 9, and two mutations with wild type).

### Survival outcome

**Figure 2:**
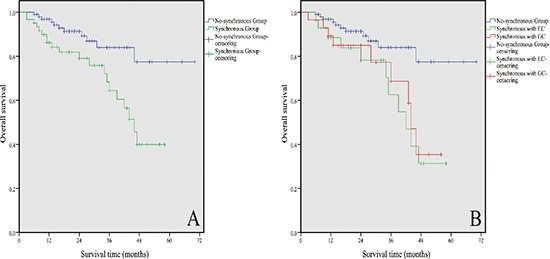
Kaplan–Meier estimates of the OS of 161 elderly patients with GISTs **(A)** OS was significantly lower in patients with synchronous digestive malignancies than that without these diseases (*P* = 0.003). **(B)** Stratification based on the types of synchronous digestive tract malignancies; the three-year OS rate of patients in the no-synchronous group was higher than that of patients with GISTs synchronous with EC (*P* = 0.005) and with GC (*P* = 0.021). GC refers to gastric cancer, and EC refers to esophageal cancer.

Thirty-one patients died for the entire cohort with a median follow-up of 21 months (range, 3–70 months). Twenty-six patients presented GIST-specific progression in the no-synchronous group. The one-, two-, and three-year progression-free survival rates in the no-synchronous group were 90.5%, 82.1%, and 66.7%, respectively. The three-year OS rate was significantly lower among patients with synchronous digestive tract malignancies than that of patients without synchronous disease (64.5% vs. 84.0%, *P* = 0.003; Figure 2A). The median survival was not achieved for patients with GISTs only (three-year OS was 84.0%) versus 24 months for patients with GISTs synchronous with esophageal cancer (three-year OS was 62.6%) and 19 months for patients with GISTs synchronous with gastric cancer (three-year OS was 68.7%). The three-year OS rate of patients in the no-synchronous group was higher than that of patients with GISTs synchronous with esophageal carcinoma (*P* = 0.005) and with gastric carcinoma (*P* = 0.021, Figure 2B). The OS of low-, intermediate-, and high-risk GISTs in the no-synchronous group were 94.1%, 89.2%, and 75.1% at three years, respectively, and no patient with very low risk died. Accordingly, OS of very low-risk GISTs in the synchronous group was 70.5% at three years. Two patients for both low and intermediate risk died, and one patient with high risk died 35 months in the synchronous group postoperatively. The univariate and multivariate analyses of factors affecting the OS of elderly patients with GISTs are summarized in Table 3. The presence of synchronous digestive tract malignancies and co-morbidity was associated with poorer OS rate in the univariate analysis (*P* < 0.05). Moreover, multivariate analysis showed that synchronous digestive tract malignancies (*P* = 0.002), mitotic count (*P* = 0.012), and co-morbidity (*P* = 0.004) were independent predictors of OS.

**Table 3:** Univariate and multivariate analysis on factors impacting overall survival in the elderly GISTs patients

Factors	Survival time (mean±SD, mo)	*P* univariate	Odds ratio* (95% CI)	*P* multivariate
Gender				
Male	26.25±17.21			
Female	24.63±15.79	0.546	1.03 (0.49−2.21)	0.931
Age, y				
≥ 75	20.09±12.44			
< 75	26.47±17.06	0.095	0.74 (0.28−1.97)	0.548
IM treatment				
Yes	25.79±16.38			
No	24.68±17.98	0.749	1.21 (0.34−4.36)	0.770
Performance status				
ECOG ≤ 1	26.12±16.64			
ECOG ≥ 2	24.87±16.69	0.639	1.97 (0.84−4.59)	0.117
Synchronous cancer^†^				
Yes	19.27±10.19			
No	28.95±15.76	0.005	9.34 (2.26−38.60)	0.002
Mitotic count				
≥ 10 mitosis/50HPF	21.29±15.87			
< 10 mitosis/50HPF	26.10±16.68	0.260	5.22 (1.43−19.07)	0.012
Tumor size				
≥ 10 cm	22.81±19.72			
< 10 cm	25.90±16.29	0.482	0.89 (0.21−3.71)	0.871
NIH risk classification				
Very low and low	27.88±15.59			
Intermediate and high	21.27±13.82	0.215	3.02 (0.77−11.85)	0.114
Co-morbidity				
Present	20.93±12.65			
Absent	28.22±18.01	0.007	3.10 (1.44−6.68)	0.004

SD, Standard deviation

* Odds ratio for multivariate analysis

CI, Confidence interval

ECOG, Eastern Cooperative Oncology Group

† Synchronous cancer refers to synchronous with digestive malignancies

HPF, High power field

NIH, National Institutes of Health

## DISCUSSION

Elderly patients with GISTs are poorly described in clinical trials. To our knowledge, this study is the first comparative analysis that considered a large amount of elderly patients with GISTs only and GISTs synchronous with digestive tract malignancies. This retrospective study primarily showed that GISTs synchronous with other digestive tract malignancies were common and occurred in almost 37.89% (61/161) of elderly patients. The difficulty in preoperatively determining asymptomatic GISTs synchronous with other digestive tract malignancies accounted for only 4.92% (3/61). Tumors with smaller sizes and very low/low risk of malignant behavior were more frequently observed in the synchronous group. Furthermore, we demonstrated that synchronous digestive tract malignancies, mitotic count, and co-morbidity were strongly associated with outcome in elderly patients with GISTs.

Increasing evidence indicates that synchronous GISTs and other neoplasms have been widely investigated in the literature. Previous studies reported that the most common GIST-associated malignancy is gastrointestinal cancer (47%), which is mainly located in the stomach and then in the esophagus [18]; this finding is in agreement with our findings. Moreover, GISTs coexisting with colorectal, prostate, pancreatic, breast, and hematological cancers have been also described. The tumor size of GISTs synchronous with other malignancies is usually smaller than 2.0 cm [3, 9, 10, 12–14, 18], and only few patients (16.7%) present a tumor size > 2.0 cm [12]. Similar to these reports, patients in the synchronous group in the present series was small with a mean size of 1.02±0.86 cm. In addition, six (9.84%) patients demonstrated a tumor size ≥ 3 cm. The proliferation rate of small GISTs is low [6]. In addition, the risk of recurrence is lower in patients with concurrent GISTs and other malignancies than in cases with GISTs only [12, 18]. A similar phenomenon was also observed in the present study. Previous studies also showed that the population of patients with synchronous GISTs and other tumors favored the elderly patients [19]. A total of 37.89% (61/161) of elderly patients with synchronous GISTs and digestive tract malignancies were observed in the present study. However, no significant difference was observed between the groups with regard to age (*P* = 0.279). Furthermore, male predominance was detected in the synchronous group. Hence, clinicians should focus on elderly male patients with digestive tract malignancies.

Clinical manifestations of GISTs are presented in different ways. Clinically overt GISTs are usually larger than 2–3 cm and present with gastrointestinal bleeding, pain, anemia, abdominal masses, and even acute abdomen in the elderly [20]. However, the majority of patients with GISTs synchronous with other malignancies are asymptomatic. Preoperative diagnosis of GISTs is particularly difficult. Lin et al. [12] reported that only one of 42 patients (2.4%) was preoperatively diagnosed with GIST coexisting with gastric cancer, and the remaining cases were incidentally found during surgery for other reasons. The present study indicated a similar conclusion, in which three patients with GISTs were preoperatively discovered in the synchronous group, accounting for 4.92% (3/61). Patients with small tumors are also asymptomatic. In patients with GISTs synchronous with other malignancies, symptoms may be concealed by the overt symptoms related to other malignancies. Thus, many GISTs may not be recognized or misdiagnosed. To prevent these conditions, medical specialists should perform imaging studies, such as computed tomography, ultrasonography, and endoscopy [21], for patients with malignant digestive tumors. In addition, Kawanowa et al. [7] demonstrated that microGISTs can be found in up to 35% of patients with gastric cancer. In consideration of these facts, the actual annual incidence of GISTs is probably grossly underestimated.

Currently, surgery is the only potential curative treatment of GISTs if radical resection is performed. However, the surgical procedure is not governed by definitive guidelines for elderly patients with GISTs synchronous with other malignancies. These GISTs lesions are usually characterized by low or very low risk of recurrence, but hospital morbidity and perioperative mortality may amount to 1% or higher in elderly patients [22]. Thus, conservative management has been recommended by some scholars [14, 23]. Nevertheless, all GISTs generally exhibit a potential for malignant transformation. A second operation for GISTs may be particularly complicated because of the severe adhesion of the abdominal cavity caused by radical resection for malignancies. Hence, GISTs tend to be removed when incidentally discovered during surgery for other digestive tract malignancies, as indicated in the study of Yan et al. [12]. Minimally invasive surgical procedures, such as endoscopic and laparoscopic resection, can be performed to decrease operative trauma in elderly patients. The recent implementation of IM in the preoperative and adjuvant setting has improved the survival of patients with GISTs. The proportion of patients with GISTs requiring IM dosage reduction seems higher for very old patients [24]. A similar phenomenon was also found in elderly patients with chronic myeloid leukemia treated with IM [25]. However, IM treatment is relatively safe and feasible for very old patients [24]. In the present cohort, drug-related adverse effects were mainly classified as grades 1 and 2. Moreover, survival was not statistically significantly different between IM and without IM treatments. This finding may be ascribed to the short IM duration with a median time of only seven months.

The possibility that the synchronous occurrence of GISTs and other epithelial tumors is only coincidental and whether the two lesions are connected by a causal relationship remains debatable. The proliferation of stromal and epithelial cells may share similar or some carcinogenic factors that contribute to their synchronous occurrence [13, 26, 27]. By contrast, some researchers reported that no relationship exists between the synchronous occurrence of these two tumors and this phenomenon is only coincidental [28]. A previous study also showed that most of the GIST cases synchronous with other tumors expressed CD117 and CD34 [14]. Notably, another study has shown that gastric GSITs synchronous with gastric cancer presented a lower positive rate of CD117 and CD34 than GISTs only [12]. This finding is inconsistent with that of the present study. Therefore, further research with a large sample size should be performed to clarify these results.

The prognoses of elderly patients with GISTs synchronous with digestive tract malignancies have been rarely discussed. Most GIST recurrence occurred within the first five years of follow-up [4]. A study reported that patients with gastric GISTs presented a 5-year OS rate of 75.9% [29], and the 5-year OS rate of patients with GISTs synchronous with gastric cancer who underwent surgical resection was 57.8% [12, 30]. The present data showed that the three-year OS rate was significantly high in elderly patients without digestive tract malignancies. This finding is in agreement with that of a previous report. Furthermore, our data showed that the OS rates of low-, intermediate-, and high-risk GISTs in the no-synchronous group were 94.1%, 89.2%, and 75.1% at three years, respectively, and no patient with very low risk died. Accordingly, the OS of very low-risk GISTs in the synchronous group was 70.5% at three years, and two patients for both low and intermediate risk died, and one patient with high risk died 35 months postoperatively. Several studies have further shown that age is not an independent predictor of OS in patients with GISTs. Moreover, patients aged higher than 65 years presented a similar outcome as younger patients [31]. Synchronous malignancies, absence of adjuvant IM therapy, and high risk of recurrence are the strongest predictors of poor OS as reported in the literature [12]. In this study, multivariate analysis showed that mitotic count (*P* = 0.012), co-morbidity (*P* = 0.004), and synchronous digestive tract malignancies (*P* = 0.002) were the prognostic factors of OS in elderly patients with GISTs.

In summary, the synchronous occurrence of GISTs with other digestive tract malignancies is common in elderly patients (≥ 65 years old). Tumors with smaller size and very low/low risk of malignant behavior are common in patients with synchronous digestive tract malignancies. OS primarily depends on synchronous digestive tract malignancies, mitotic count, and co-morbidity. However, this finding should be carefully interpreted because of its retrospective nature. Moreover, the existence of interactions among the tumors was not determined. Hence, further research based on large populations is required to elucidate the association between dual tumors.

## MATERIALS AND METHODS

### Patient selection

The inclusion criteria are as follows: (1) Histological diagnoses of all primary GISTs were confirmed in the Department of Pathology at the West China Hospital, Sichuan University. (2) Patients aged ≥ 65 years old. (3) Patients with complete medical records. The exclusion criteria are as follows: (1) Patients were excluded if they had malignancies other than malignant digestive tumors with GISTs. (2) GISTs diagnosed as recurrent or metastatic. Accordingly, 161 cases were retrospectively selected from 569 cases with GISTs. These cases were consecutively collected from January 2009 to June 2014, from the database of the West China Hospital, Sichuan University. Of these 161 patients, 61 were diagnosed with GISTs synchronous with digestive tract malignancies (synchronous group), whereas 100 were diagnosed with GISTs only, and none was diagnosed with other tumors after the initial GISTs diagnosis (no-synchronous group). The Institutional Review Board and Ethics Committee of the West China Hospital of Sichuan University informed that an ethical review was not needed for this retrospective study.

### Surgery and medication

Patients with GISTs or digestive tract malignancies underwent surgical treatment with curative intent. Operation consent was obtained from each patient who underwent surgical resection in this cohort. Surgical procedures were variably performed according to tumor size, location, and types of cancer; these procedures include subtotal gastrectomy, total gastrectomy, transhiatal esophageal resection, abdominoperineal resection, and endoscopic submucosal dissection. Lymphadenectomy was routinely performed for malignant alimentary tract tumors. The risk stratification of GISTs was evaluated according to the modified National Institutes of Health (NIH) classification [15]. Patients with GISTs with large tumor size (R0 resection cannot be obtained) or GISTs that coexist with multiple distant metastases at the time of diagnosis were treated with imatinib mesylate (IM, Glivec^®^/Gleevec^®^, Novartis Pharma AG, Basel, Switzerland) as preoperative therapy. Patients who received preoperative IM therapy were confirmed to have GISTs through aspiration biopsy according to the National Comprehensive Cancer Network guidelines [16]. The optimal response to treatment was evaluated on the basis of the Choi criteria [17]. The adjuvant IM treatment for intermediate- and high-risk patients and for cases without radical resection was administered by the attending clinicians after securing patient's consent. The suggested IM dosage was 400 mg per day.

### Clinicopathological data collection

All clinicopathological data were retrospectively reviewed from medical charts. These data included demographic data (age and gender), length of hospitalization, tumor site and size, clinical presentation, pathological parameters (mitotic index, mutation status, morphological variant, etc.), IM duration, drug toxicity (evaluated on the basis of Common Terminology Criteria for Adverse Events version 3.0), Eastern Cooperative Oncology Group (ECOG) performance status, and analysis of immunohistochemical staining (CD117, CD34, Dog-1, S-100, SMA, and desmin). The reported co-morbidities included chronic pulmonary disease, diabetes mellitus, cardiovascular and cerebrovascular diseases, chronic liver, and renal diseases.

### Survival analysis and follow-up

Overall survival (OS) was defined as the time from the surgery to the date of death or last contact with patient. Progression-free survival in patients in the no-synchronous group was determined from the start of any treatment until disease progression. To date, determination of GIST lesions from recurrence/metastases of known digestive tract malignancies remain difficult under some conditions. Thus, GIST-specific progression-free survival analysis between the two groups was not performed. Follow-ups were conducted through office visit, telephone call, or outpatient clinic visit from November 2014 to December 2014.

### Statistical analysis

All statistical analyses were performed using the Statistical Package for the Social Science (SPSS), version 17.0 for Windows (SPSS Inc, Chicago, IL, USA). Measurement data were expressed as mean ± standard deviation. Differences between groups were analyzed using analysis of variance for continuous variables and χ^2^ test or Fisher's exact test for categorical data. Cumulative survival was determined using the Kaplan–Meier method and log rank test. Univariate and multivariate analyses were used to explore independent prognostic factors by Cox regression. Differences with two-sided *P* < 0.05 indicated statistical significance.
